# Automatic Detection of Colorectal Polyps Using Transfer Learning

**DOI:** 10.3390/s21175704

**Published:** 2021-08-24

**Authors:** Eva-H. Dulf, Marius Bledea, Teodora Mocan, Lucian Mocan

**Affiliations:** 1 Department of Automation, Faculty of Automation and Computer Science, Technical University of Cluj-Napoca, Memorandumului Str. 28, 400014 Cluj-Napoca, Romania; Marius.Bledea@student.utcluj.ro; 2 Department of Physiology, Iuliu Hatieganu University of Medicine and Pharmacy, 400349 Cluj-Napoca, Romania; teodora.mocan@umfcluj.ro; 3 Nanomedicine Department, Regional Institute of Gatroenterology and Hepatology, 400000 Cluj-Napoca, Romania; 4 Department of Surgery, 3-rd Surgery Clinic, Iuliu Hatieganu University of Medicine and Pharmacy, 400349 Cluj-Napoca, Romania; lucian.mocan@umfcluj.ro

**Keywords:** colorectal cancer, computer aided decision support system, artificial intelligence

## Abstract

**Simple Summary:**

Colorectal cancer represents one of the major health problems due to high incidence and mortality rates. A diversity of treatment options as well as a rising population require novel diagnostic tools. The main goal of the research was to develop a novel complex colorectal cancer decision support system based on artificial intelligence. The developed system can classify eight classes of tissue and can identify the malignant areas. In order to allow the easiest and most intuitive interaction with clinicians, the corresponding application was also built.

**Abstract:**

Colorectal cancer is the second leading cause of cancer death and ranks third worldwide in diagnosed malignant pathologies (1.36 million new cases annually). An increase in the diversity of treatment options as well as a rising population require novel diagnostic tools. Current diagnostics involve critical human thinking, but the decisional process loses accuracy due to the increased number of modulatory factors involved. The proposed computer-aided diagnosis system analyses each colonoscopy and provides predictions that will help the clinician to make the right decisions. Artificial intelligence is included in the system both offline and online image processing tools. Aiming to improve the diagnostic process of colon cancer patients, an application was built that allows the easiest and most intuitive interaction between medical staff and the proposed diagnosis system. The developed tool uses two networks. The first, a convolutional neural network, is capable of classifying eight classes of tissue with a sensitivity of 98.13% and an F1 score of 98.14%, while the second network, based on semantic segmentation, can identify the malignant areas with a Jaccard index of 75.18%. The results could have a direct impact on personalised medicine combining clinical knowledge with the computing power of intelligent algorithms.

## 1. Introduction

Colorectal cancer (CCR) is the second leading cause of cancer death and ranks third worldwide in diagnosed malignant pathologies (1.36 million new cases annually) [1], which provides an important and extensive source of information to help medicine advance. It represents 10% of the cancer mortality rate each year [2]. Unfortunately, based on the data collected so far, it is estimated that the number of patients affected by this disease will increase considerably in the years to come. One of the main causes for the increasing incidence of the condition is the ever-increasing rate of ageing of the population. In addition, cancer-related death has increased by 45% in recent years and there is a high chance that the rate will rise to 60% over the next 15 years [2]. It is estimated that the market value for colon cancer therapies will reach as high as $11 billion by year 2025 due to the increase of branded therapies.

CCR is a condition that alters malignant cells inside the colon or rectum [2]. In the diagnostic process can be observed also benign cells, but these do not pose a real danger, not being favorable to the tumor [2]. Polyps—the abnormal growth of unhealthy cells—can be either benign or malignant [3]. The benign ones are not harmful, but they can easily turn malignant in the future, requiring immediate further investigation. Polyps are divided into two categories: adenomatous polyps, leading to the disease itself, and hyperplastic polyps, occurring with a higher frequency, and generally not considered to be a sign of disease [3]. The colorectal cancer can also spread to other organs in close proximity, for instance: the bladder, prostate or uterus.

Regarding the success of a treatment, even it is of a surgical nature or neoadjuvant method (chemotherapy, radiotherapy, hormone therapy), the faster the disease is diagnosed, the higher the chances of healing. Given that a diagnosis is difficult to establish by humans due to the multiple factors involved, it is the right time when exact sciences can improve and streamline this process.

The proposed solution aims to support doctors who are currently establishing the diagnosis manually. The proposed computer aided diagnosis system analyses each colonoscopy and provides predictions that will help clinicians to make the right decision. This whole process is ensured in an exhaustive manner, requiring a complex analysis of data, leading to a result that will be communicated to the patient. In the current form such analysis is made by clinicians. It is certainly difficult to take all that information into account and to establish a result with great accuracy. In addition, human error occurs due to various factors having an impact on doctors. To reach a more precise diagnosis, many specialists should meet, analyze and agree on common grounds. Obviously this method is not feasible in everyday life. What if every doctor had all the knowledge of specialists at hand without requiring their physical presence? It would certainly help and increase the success rate of the resulting diagnoses by decreasing human error. The proposed solution aims to rely also on previous colon cancer cases, which have been carefully examined and to which we have the certainty of diagnosis. In other words, past cases are situations where a diagnosis has been made, and with time, it has also been confirmed whether the decision made was correct or not. This data can serve as a solid research foundation, in the direction of identifying the essential factors that trigger this pathology.

## 2. Related Works

The tool used to achieve the aforementioned goal is artificial intelligence. There are a number of approaches based on machine learning techniques in the medical field, with a focus on colon cancer, but also other types of cancer, each of them containing materials and information relevant to the design of intelligent algorithms.

In paper [3] the authors describes the machine learning method applied to classify histological images and identify the cancerous areas. They used two deep convolutional networks. The first is necessary for the separation of the background from the glands, and the second identifies the different structures of the glands, dividing them into two categories: malignant or benign. The architecture of the two convolutional neural networks was inspired by the classic LeNet-5 network, first developed in 1998 by LeCun et al. in [4] and originally designed to recognise handwritten figures. The used network contains seven layers: four convolutional filtered and three fully connected layers. ReLU functions are used as activation functions throughout the network. Concerning the training process, the random mini-lot method with gradient drop, with 200 images per epoch, was used until the stopping criteria were met. The used data set consisted of 165 images containing properly labelled malignant and benign tissues. The results obtained were over 95% accurate.

In [5] it was demonstrated that the aggressiveness of colorectal cancer is directly influenced by gland formations. Therefore, in order to have the most relevant classification of different types of tumor, it is important to distinguish the glands from the other structures that appear in a histological image.

Xinghazi Yue et al. [6] came to the conclusion that there was a significant increase in the algorithm’s performance when the concept of transfer learning was applied. By using this concept, the training becomes much faster and the predictions become more accurate, in comparison to training the network from scratch. The parameter values are chosen in such a way that the early layers manage to learn the more general characteristics and the deeper layers focus on the characteristics specific to the problem in question [6]. The more general traits are: edges, contour, shadows, and in terms of specific characteristics: types of tissue, cells, polyps. Reference [6] also discussed the possibility of predicting the chances of survival of at least 5 years after surgery, analyzing histological images. They applied the principles of Reinhard normalisation [7] and the principal component analysis (PCA) method in order to reduce the dimensionality of the data set by preserving the most relevant characteristics. The authors of work [8] applied the idea to use normalized images instead of high resolution original images, which is hard to be processed. They proposed removing from the dataset the regions containing less than 30% tissue or having excessive blood. To reduce the probability of the system being over-trained, the data augmentation concept can be applied to the data pool. This involves applying geometric operations, such as turning 90 degrees, horizontal or vertical reflections, or applying blurred Gaussian filters [6]. The purpose of these operations is to help the algorithm become invariant to different rotations or shades of the same regions, to be able to focus on what is important, without waiting for the data set to be perfectly served [8]. A non-supervised learning method was used for the zone classification part because there was no set of labels that could have corresponded to well-defined classes, and the human work required to do so would have been too costly. The k-means-cluster algorithm was used, which operates by the following principle: k cluster centroids were chosen and points were assigned to them, choosing the centroid closest to that point. To increase the robustness of the algorithm, different randomly initialised parameters were applied and continued with the set that generates the smallest loss [8]. The structure of the chosen network by Yue et al. in [6] was inspired by the well-known VGG16 and adapted to fit the current requirements of the problem. VGG16 is a convoluted network developed by K. Simonyan and A. Zisserman in [8] that managed to reach an accuracy of 92.7% on a data set of 14 million images, belonging to 1000 distinct classes. The conclusion reached in [6] is that five clusters are the most suitable and easily to interpret model: Cluster 0: out of tissue; Cluster 1: contains red blood cells; Cluster 2: contains cancer cells and antibodies; Cluster 3: empty; Cluster 4: contains fat. The authors aim is to predict the lifespan of patients without requiring prior work by doctors to prepare a database of appropriate images and labels [6].

The idea that a pre-trained network produces superior results when analyzing medical images, even if the network initially did not come into contact with such data, is also reinforced in paper [9]. G. Urban et al. aimed to identify polyps in the patient colonoscopies. Unlike previous works, [3,6], in which histological images were analyzed, in [9] the object studied was a set of images captured in real time during the colonoscopy process. Thus, the authors applied the concepts of supervised learning, using deep convolutional networks, with randomly initialised parameters, but also with parameters already calculated after training on other data sets. They also tried different architectures whose foundations were similar in many aspects: convolutional layers, densely connected, dimensional reduction, non-linear activation functions (RELU) and normalisation operations. The main difference was made at the last layer, when it was adapted according to the desired result: binary classification or framing in a rectangular form of polyps. In the second case, being a regression problem, the performance criterion was quantified using the L2 function (root mean square). While for the first binary classification situation, the authors used the Kullback–Leibler criterion [10] (assess the percentage of lost information using an approximation). To reduce the chances of the model becoming over-fitted, the authors introduced dropout layers in addition to the original structure. The purpose of these layers was to deactivate neurons in a non-predictable way, with the probability of 0.5 which translates as the probability of each neuron being disabled at each iteration is 50% [11]. Studies have also shown that the introduction of these layers improves the system robustness and eliminates dependencies between neurons [11].

A data augmentation procedure was also successfully applied in [12] in which, using deep convolution networks, the analysis and localisation of the brain ventricles of a new-born was intended. The volume of data was not very extensive, hence the augmentation process was the best method to increase system performance. Paper [12] describes the different types of augmentation and their impact on the results. The fields that provided the significant improvement to the algorithm are highlighted: the horizontal flip offers a score quantified by the dice loss criterion [13] ≥0.8 and a *p* value [14] ≤0.05. It is most likely that the reason is that these transformations create images similar to the real ones, unlike other methods that produce improbable data.

An aspect worth mentioning is that images containing medical utensils such as scissors, string, scalpel and also pigmented tissues, were intentionally inserted into the data set, both in the images in which the polyps were present and in those in which they were not. The reason was to eliminate the possibility of the model associating the presence of utensils with that of polyps or vice versa [9]. In terms of the data set, a multitude of materials usable for training were made available [15].

For the detection of polyps and their framing in rectangular forms, three cost functions are usually used: root mean square error, dice loss criterion [13] and a variation of the algorithm “YOLO” (You only look once) [9,16]. The latter algorithm is revolutionary in terms of the field of object detection in images.

In [9] different architectures of neural networks were presented. They were distributed into two categories: those previously initialised (PI) and those that were not (NPI). The first category, PI, was previously trained on the ImageNet data set [15], which comprises approximately one million images grouped in more than 1000 classes. For previously trained networks, the well-known architectures were VGG16 [17], VGG19 [18] and ResNet50 [19]. In the case of previously uninitiated parameters, the networks were trained directly on the image set of interest. The authors of [9] concluded that the presence of polyps can be detected with an accuracy of 96.41% using convolutional neural networks that were previously trained on a general set of images and then on the desired set for the specific problem. This inference was also confirmed in [3,6].

In [20] the authors proposed a method based on the architecture of the Inception Resnet network with the objective of the detection of polyps in medical images. Using this structure, they managed to achieve high performance compared to the applications in [21,22], where the transfer learning concept was also used. They also focused on data augmentation and its impact on model performance. Training a convolutional neural network requires a large amount of data to deliver satisfactory results. As an example, the AlexNet model [23] was developed and trained using a database of 1.2 million images to be able to successfully identify different objects. Extrapolating for the situation where the purpose is to classify tissues and polyps, the need for a huge database is mandatory to produce satisfactory results. Therefore, to overcome this impediment, data augmentation has been applied to increase the amount of information.

A different approach is presented in [24], where the authors identified that the detection of small polyps is very difficult. They thought of applying different percentages of enlarging and shrinkage (three zooms-out and a zoom-in). This allowed the model to detect areas with reduced surface polyps. The network architecture was based on the “region-proposed” model. Usually, the last layer of convolution in a deep convolutional network is used as an input of the “region-proposed” model [24].

As it is also concluded in [3,6,24], the use of previously trained networks on large data sets and subsequently on the smaller set available for the specific problem, provides results that outperform an identical architecture that has randomly initialised parameters.

Image processing in the medical field is also used with great results for other types of image. For example in [25,26] a diffusion-weighted magnetic resonance imaging is discussed for breast and lung cancer detection. Reference [27] presented a survey of deep learning algorithms used in medical applications.

The novelty of the present work is the development of a computer-aided diagnosis system, including both offline and online image-processing tools. The methods used exclude all the aforementioned disadvantages. The network for the diagnosis system was trained on the Microsoft COCO dataset [28] (Common Objects in Context) which consists of 122,000 images and 90 different common objects: people, bicycles, animals, cars. On the other hand, for the training phase of the model, the CVC-CLINIC dataset [29] was used with only 612 images containing polyps and the same number of images indicating their position. In order to address a bigger number of images, a unique augmentation was performed, consisting in a greater range of operations applied to data: tilts along the axes and zoom-in or zoom-out, various luminosities added that help the model become invariant to certain areas that can be covered by the patient’s utensils or other organs, thus creating shade.

This paper is structured in four sections. After this introductory section, Section 2 presents the materials and methods used, while Section 3 presents the results obtained. The work ends with a concluding section.

## 3. Materials and Methods

The goal of this research is to build an application to help medical professionals fight colon cancer, both in the detection and post-operative phase. Pursuing this goal, as a starting point, a database to start with network training is needed. The chosen data set is called ‘Kvasir’ [30] which is a database made available to the public free of charge for research purposes. The images were taken using endoscopic medical equipment in four hospitals in Norway on 470,000 patients. Then all this data were carefully labelled by the endoscopic specialists. The total number of classes in which the data are divided is eight. There are three classes of pathological findings type, three classes of anatomical landmarks type, and two classes that show areas of extirpated polyps [30].

In the following are presented the medical meaning of the aforementioned classes in order to understand their importance for colorectal cancer.

❖ Anatomical landmarks: are areas of the gastrointestinal tract easily visible through the endoscope. They are used to indicate how far the video camera has reached through the colonoscopy process.
  - Z-line: marks the transition between the oesophagus and the stomach. It is of interest because it indicates whether or not the disease exists because this is the area where signs of gastroesophageal reflux may appear [30].
  - Pylorus: is the area under the opening of the stomach to the duodenum. It has the muscles involved in transporting food from the stomach. Identification of the pilor is necessary for the detection of ulcers, erosions and stenosis [30].
  - Cecum: is the most proximal part of the large intestine. The successful completion of the colonoscopy up to this point can be interpreted as an indicator of quality [30].
❖ Pathological findings: in this context of endoscopies, pathological findings refers to abnormal entities that occur in the gastrointestinal tract. These may be signs of an ongoing disease or one that is about to begin. Their detection is extremely important and especially relevant for initiating the suitable treatment [30].
  - Esophagitis: is an inflammation of the oesophagus visible as a rift of the oesophageal mucosa in relation to the Z-line. This is most often caused by gastric acid that returns back to the oesophagus. Clinically, early treatment is necessary for the improvement and prevention of possible complications [30].
  - Polyps: are lesions of the intestine detectable as prominent areas of mucosa. They may be elevated, flat or pedunculated. Most polyps are harmless, however a small ratio of them can cause colorectal cancer. It is obvious then that their detection and elimination reduces substantially the risk of developing the disease. They are often missed by the human eye within colonoscopies, a gap that is intended to be covered by artificial intelligence [30].
  - Ulcerative colitis: is a chronic inflammatory disease affecting the large intestine. It mainly affects the quality of life. Therefore, it is not a disease directly related to colorectal cancer, but investigations to detect cancer can also lead to this adjacent disease [30].
❖ Polyps located in the large bowel: are the precursors of cancer so they must be removed as soon as possible. One such technique is mucous endoscopic resection. This involves injecting a liquid under the polyp, causing it to detach from the tissue under it, and then removal using surgical scissors.
  - Coloured polyps: are those polyps on which the solution has been applied that causes them to detach from the tissue and at the same time colours them with a bluish hue. The edges are visible and the surrounding healthy tissue is distinguished. They are of interest because they reveal the success of the extirpation or any remaining malignant areas [30].
  - Dyed resection margins: are those areas left over from the elimination of cancerous areas, which indicate whether the polyp has been completely removed or not. The remaining tissues may continue to develop and thus the disease returns [30].

Following the papers [3,4,9,20], it has also been decided to apply the transfer learning strategy in this paper. We chose some of the most important models that operate with image data. The focus of this paper is to develop an application capable of helping the doctors by automating the process of polyp detection and identification. Relying on the Kvasir [30] dataset and the advice and guidance provided by the clinicians involved in present research, we aim to enhance the detection rate of colorectal cancer.

For the consistency of the results and the possibility of comparing algorithms, we used the same parameters in terms of the training process for all the networks [31]. The method chosen for optimisation was stochastic gradient descent with momentum, where the value of the contribution of the previous step, momentum, was 0.9. The total number of epochs was 10, the learning rate was $3\cdot 10^{-4}$, and the data were randomly mixed at the beginning of each era. Thus this avoided the model becoming over-trained by learning a specific data sequence and in addition due to the optimisation method, which was based on the principle of finding the minimum by choosing random values, increasing the training speed. The parameters performed in Matlab^®^ are listed in Table 1.

Batch size (miniBatchSize) was chosen as 10. It was a sub-set of the training set and used to evaluate the gradient and modify parameters accordingly [31], after all 10 images were propagated forward through layers. Generally, a suitable value was desired, which was as far away from 1 as possible because too small batches require too many calculations and in addition the model tends towards over-training. On the other hand, the high values overload the available computational resources and then become impractical. In this particular case, a balance was found by using the value 10. Validation data were used for the periodic determination of progress and also use as a criterion for stopping training [32]. The last two arguments refer to the display of real-time data on the screen as the algorithm progresses.

### 3.1. Image Classification

The first steps for developing the comparative study between network performances was to prepare the algorithm to automate the process, without having to require significant changes for each new architecture. The function called “FindLayersToReplace” is used, which identifies for each particular case which layers need to be replaced. In most situations the last 3 layers are replaced, with small exceptions where only the last 2 require modifications.

**Figure 1:**
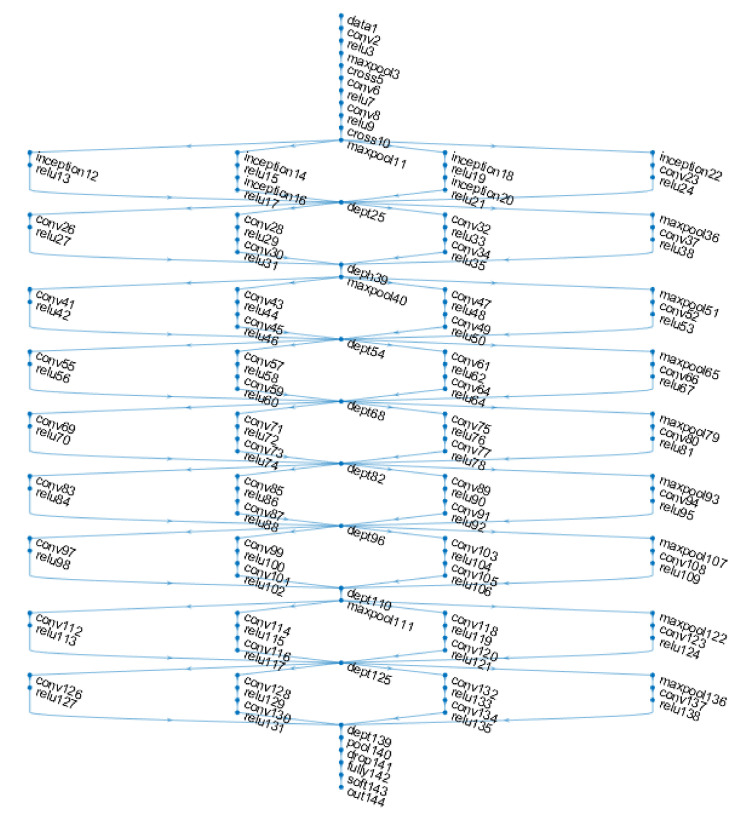
Google Net network architecture [30].

The work is started by using the network “GoogleNet” [33], the winner of the competition “ImageNet 2014” [15]. This network was trained on the set of images provided by the organisers of the contest [15] which contains over 1.2 million images, 1000 categories, 50,000 images for validation and 100,000 for testing. The idea behind the network architecture is the concept called “Inception Layer” and involves gathering information from a wider area without omitting the details. This becomes possible using the Gabor filter theory [33] with different dimensions. Gabor filters are used in order to detect complex contour detections in images. The model contains a number of 22 layers, each made up of activation and dimensional reduction functions resulting in a number of 144 components. Figure 1 shows the network architecture. Inception layers are identifiable by the 9 identical branches that occur along it. Such a fork is composed of 4 branches with three different convolution layers, in terms of filter size, and one sampling layer.

Initially the network was designed to recognise 1000 categories [33]. In the present work the network was modified to identify only eight possible classes, according to the current problem. It should be pointed out that two versions of the network exist. The first is the one in which the network was trained on the data set “ImageNet” [15] and the version in which it was trained on the set “Places365” [34] which consists of 365 categories and 1.8 million images.

Another popular and common architecture in image classification problems and used here is AlexNet [24]. It is also able to distinguish 1000 different classes. The images that can be served as an input must be 227 × 227 × 3 in size, the last argument meaning that all data must be colour. This consists of five convolutional layers and three densely connected [24]. The activation function used after each convolution layer is RELU [35]. A number of 62.3 million parameters make up the layers of the network and require a training of 90 epochs, which takes approximately 5–6 days [24]. Convolutional layers decrease as depth increases, from 11 × 11 to 5 × 5 and eventually to 3 × 3. Before the last layer, a deactivation function (Dropout) [24,31] is introduced to prevent over-training. Each neuron has a 50% probability that it will be excluded from the back and forth propagation process at a given iteration.

The third architecture to be tested was VGG16 and VGG19 (Visual Geometry Group) [24]. The difference between the two is the additional number of layers that the latter has. The numeric suffix signifies the number of layers. As input it supports RGB (colour) images with a size of 224 × 224. These are passed on to 3 × 3-sized convolutional layers (the smallest possible size able to detect top/bottom left/right zones [24]). The number of filters increases as the depth increases, from 64, 128, 256 and finally 512. The activation functions used are RELU [35] just as in the GoogleNet and AlexNet architecture [24]. The main disadvantage of this type of network is the extremely long training time required. The number of parameters is very high, so it requires significant computational effort, and the lack of random deactivation layers (Dropout) makes each neuron to be taken into account in the process of spreading the error backwards [36]. VGG19 consists of 16 convolutional layers and 3 densely connected, while VGG16 has 13 convolutional layers and as many densely connected as VGG19 [24].

A zero-centred normalisation of the pixels of each image and variation of 1 is applied to the data by subtracting the mean value and dividing by the standard deviation. This operation allows the data to be compared with each other and increases the speed of the training process due to the fact that they no longer have to perform operations with large numbers [11].

Another tested architecture is InceptionV3 [37]. It is based on the GoogleNet model [33], which is also known as InceptionV1. As the suffix suggests, this is an improved version. The premise from which Chr. Szegedy started in [37] was to reduce the degree of “strangulation” of the network imposed by the convolutional layers. Reducing the initial size of images results in loss of information. Therefore, the proposed method to overcome this drawback is the factorisation of the convolutive layers. This technique reduces complexity and implicitly the training time, while managing to maintain the efficiency of the model. The 5 × 5-size convolution filters have been replaced by two filters of 3 × 3 dimensions. If it initially had 5 × 5 = 25 parameters, after applying factorisation it will be reduced to 3 × 3 + 3 × 3 = 18 parameters for the two filters, thus reducing by 28% the total number of variables. In addition, asymmetrical convolutional factoring is applied, a method with the same benefits. The 3 × 3-sized filters, consisting of 9 parameters, will be optimised by using two filters with dimensions 3 × 1 and 1 × 3, so the resulting number of parameters will be 3 × 1 + 1 × 3 = 6. This method offers a mitigation in the number of parameters by another 33%. The InceptionV3 contains 42 layers, requires computational effort of more than 2.5 times, but is more efficient than the VGGNet model.

### 3.2. Polyps Identification

To identify malignant regions, also known as polyps, from images containing such regions, semantic segmentation process is used. This is also a data classification technique, but unlike the previous case when the classification was carried out on the entire image, i.e., on all the component pixels, this algorithm assumes that each pixel has a label that corresponds to it, so the classification will be made pixel by pixel.

**Figure 2:**
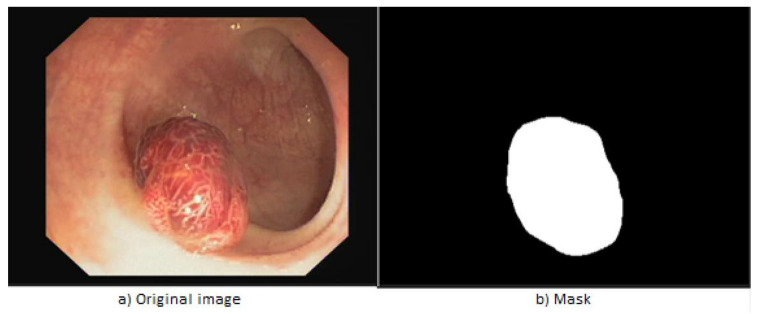
Example extracted from “CVC-ClinicDB” [38].

The first step in developing the algorithm is the data acquisition. The “CVC-ClinicDB” dataset [38] is used, which is made available free of charge for research/educational purposes. Thus, the resources consisted of 612 photographs extracted from endoscopies taken by doctors from the Clinical Hospital, Barcelona, Spain, and another 612 masks that were developed by members of the Department of Computer Vision, Barcelona, Spain [38]. The latter are black-and-white images where the area containing the polyp is highlighted with white, and with black the healthy tissue. The dimensions are 288 × 384 × 3 pixels for the first dataset mentioned, and for the other one with mask images they are 288 × 384 × 1. Figure 2 shows an example of a pair of images, in which (a) is a screen capture made from an endoscopy, and (b) is the related mask that makes it possible to differentiate the polyp from the rest of the tissues.

Since semantic segmentation assumes that each pixel in the image has a class associated with it [39], the data in the “CVC-ClinicDB” dataset [38] is also required to comply. Initially the pixel values that make up the array, essentially the image, had values in the range [0, 255]. Taking this into account, the values were changed in order to have the set of possible values with only two elements, since the goal is to classify two types of pixels: tissue and polyps.

With this operation, the “tissue” label is assigned to the value 0, and label “polyps” to the value 255, using the *pixelLabelDataStore* function. The function receives as parameters: the location of mask images, the classified classes and the pixel values corresponding to each class respecting the order in which they are entered in the class vector. The class vector is defined in Equation (1) and the pixel values corresponding to each class are represented in Equation (2).

(1) $$classes=\left[\begin{array}{c} tissue\\ polyp \end{array}\right]$$

(2) $$pixel\_value=\left[\begin{array}{c} 0\\ 255 \end{array}\right]$$

The starting point is the architecture of the network “ResNet18” [40]. It is originally designed for image classification, so a few changes are needed in order to classify pixel by pixel. It is proven that deep convolutional networks provide a conducive development point in the creation of segmentation algorithms [40]. Most often, as in this case, the original architecture is preserved until the last convolutive layer. It is considered that up to that point the model has extracted the important characteristics of an image.

**Figure 3:**
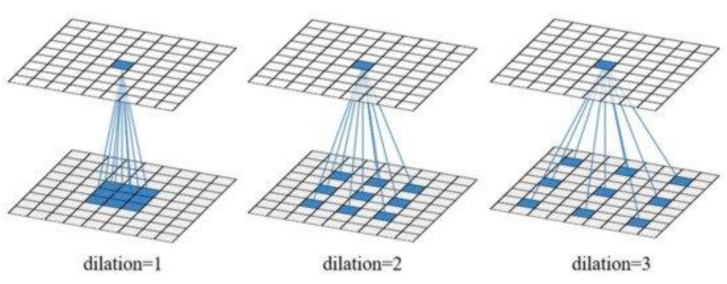
Expansion rate impact on convolutional filters [41].

The element called “encoder” introduces a new concept of expanded convolution. For improved feature capture, this type of convolution, described in Equation (3), is introduced, and helps the decoding part where the images are brought back to their original size and superimposed over regions identified by the model:

(3) $$y\left[i\right]=\underset{k}{\sum }x\left[i+r\cdot k\right]\;\cdot \;w\left[k\right]$$

where ***y***—resulted value, ***x***—original value of the pixel, ***w***—filter value and *r* expansion value. For r=1 it is obtained the normal convolution. In Figure 3 can be observed the impact of expansion rate *r* on filters.

The present study used the filters with expansion factors of: 1, 6, 12 and 18, according to studies conducted by Chen [40] which showed that these values provide optimal results.

The removed layers are the last five, those involved in the classification. Instead of these eliminated layers, expanded convolution additional ones are added, followed by the *decoder*. This consisted of six convolutional layers, five ReLU activation layers [31] and two transposed convolution layers. The latter has the opposite effect of convolution, i.e., the return to its original dimensions.

**Figure 4:**
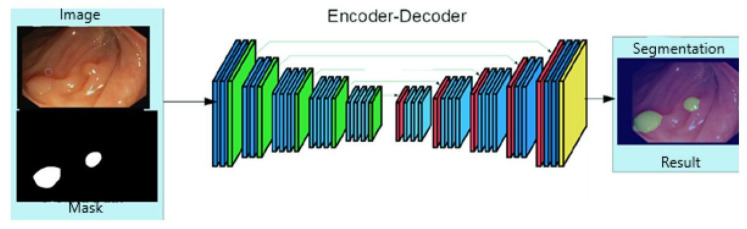
ResNet18 model architecture [39].

Figure 4 [39] describes the architecture of the model used for image segmentation in the training process. It is noticed that the provided images are those that want to be segmented and the corresponding masks As a result the overlap of these two will be obtained. If testing is performed, respectively the segmentation of new data, there will be no mask as input to the process. The model will propose a region of interest and will overlay it with the original image.

An important aspect that influences the model’s ability to identify the regions of interest as accurately as possible is to consider the proportion between pixels labelled as polyps and those classified as tissue.

**Figure 5:**
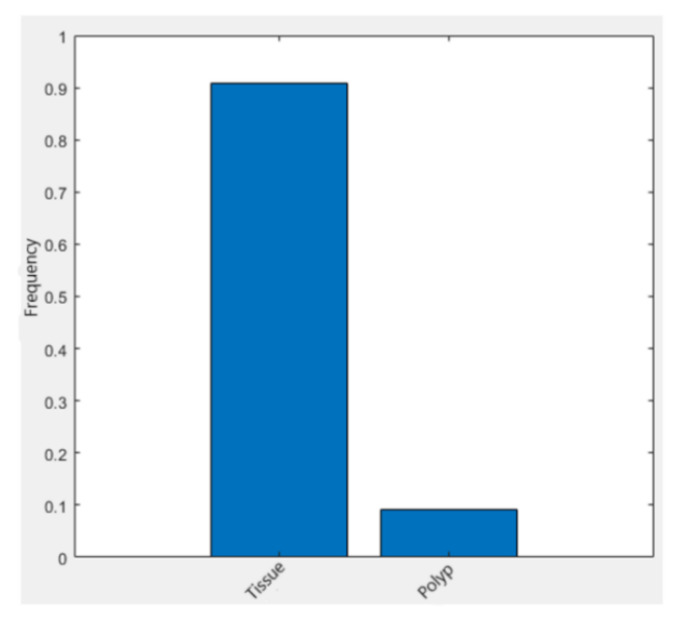
Pixels frequency corresponding to each class.

As seen in Figure 5, the dominant pixels are the tissue type and are 90%. Thus, in order to balance their influence in the training process and to prevent the model from classifying only tissues, weights were assigned. Equation (4) presents the calculation for the frequency of each class, while Equation (5) the method for calculating the weights of the classes.

(4) $$\nu_{class}=\frac{Number\;of\;Class\;Pixels}{Number\;of\;Total\;Pixels}$$

(5) $$Weight=\frac{average\;of\;each\;\nu_{class}}{\nu_{class}}$$

In the case of polyps, a weight of 5.45 is used, and for tissue the weight is 0.55. Thus the importance of the data has been balanced, and the model is able to classify each pixel correctly.

Regarding the training part, the method of decreasing the gradient with momentum is chosen as the optimisation method, the initial learning rate 10^−3^, reduced by 30% at every 10 epochs. By doing so, the model will learn at a faster pace at the beginning, and as the parameters approach the values needed to converge to the local minimum, the learning rate will decrease to approach the minimum as much as possible, without oscillations around the minimum. Momentum is the contribution of the previous step to the change in value at the current step. Thus, a value of 1 represents a maximum contribution, and a value of 0 a zero contribution to the current step. Validation data were also provided to assess the progress made with a cadence of 50 iterations and a condition for completing the training process through validation (validation patience) was introduced. If after five iterations the error on the validation set remains constant or increases, then the training process is suspended. The maximum number of epochs chosen is 40 and in addition, at each new epoch the data are shuffled randomly. The reason behind this choice is to eliminate the possibility that the model learns the order in which the data are served and thus over-training occurring.

Table 2 shows the parameters of the Matlab^®^ “trainingOptions” function. This returns an object delivered for the “trainNetwork” function in order to use the options during training. The training function receives as parameters: the dataset as a “PixelLabelImageDataStore” object, which contains the original images and the corresponding masks, the network architecture as a graph and the options shown in Table 2.

Following the call of the “trainNetwork” function, the training process will be launched where information such as: accuracy, error, elapsed time from the beginning of the process, learning rate and number of iterations will be available for viewing.

The data set was segmented into three parts respecting the proportions 60/20/20. We selected 60% of the total number of images for training, 20% for validation during the training process, and the last part was reserved for evaluating the model’s performance after training. Given that the dataset [38] consisted of screenshots taken from endoscopes, there were numerous sequences of images that showed the same area with small differences in position and hue. Thus, in order to have a more robust model, image indices were random. Images were extracted from different areas of the set, beginning, middle or from the end to avoid contiguous locations. In this way, the data were properly distributed for each subset (training, test, validation) separately.

Due to the conditions under which colonoscopies are performed, the images usually have a variety of features that diminish the quality of the data and hinder the model’s ability to predict malignant areas. These features include: brightness, how close or far from the polyp the camera is, their location, the colour of the areas, or the angle of filming. Given all these impediments, augmentation methods were used for the data set, for example rotation by 90 degrees, applying the cut along the x-axis, magnification or reduction of the original image, applying the cut along the y-axis. In addition, the number of available data were relatively small, so the increase brought another benefit, namely the generation of new valid data for the training process. In this way, horizontal and vertical mirror transformations, rotations with angles between [0, 90] degrees, cuts along the *X* or *Y* axes, displacements along the axes and increases or decreases are applied. All these values were randomly generated and were in a normal distribution of zero mean with the specified lower and upper limits, respectively. Each filter had a 0.5 probability of being applied to an image. Following these operations, the model should become invariant to images that contain the regions of interest in a distorted form. In general, datasets are carefully chosen and processed, so there are no more general images such as some screenshots taken from a video. These operations were implemented using the function in the Matlab^®^ “imageDataAugmenter”, with the parameters presented in Table 3.

**Figure 6:**
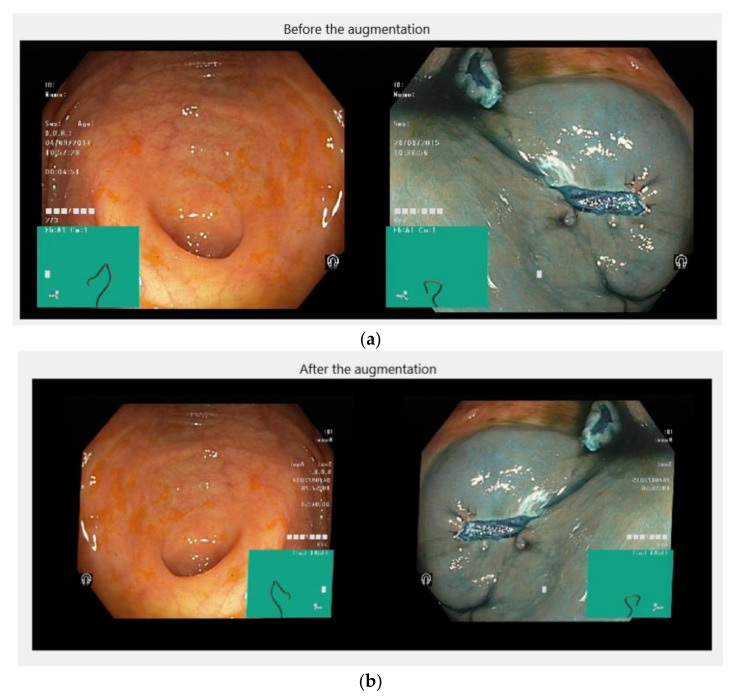
(**a**) Before and (**b**) after augmentation.

Figure 6 shows two examples of images on which the operations of rotation, scaling, reflection and cutting were applied.

### 3.3. The Developed Application

The user interface was developed using Matlab^®^ “App Designer” provided by MathWorks. The goal was to keep it as intuitive as possible, because the end user of the application was not a specialist in computer science. The main components of the application are:

- A “Button” object to select and load image data from a storage device;
- An “Image” object to view uploaded images;
- A “Table” object to display information such as: image source, network prediction, and confidence percentage on the prediction made;
- A “Camera” object with which the user can connect to the available camera and perform an online endoscopy process;
- Two variables in which the networks are loaded at the time of application launch.

**Figure 7:**
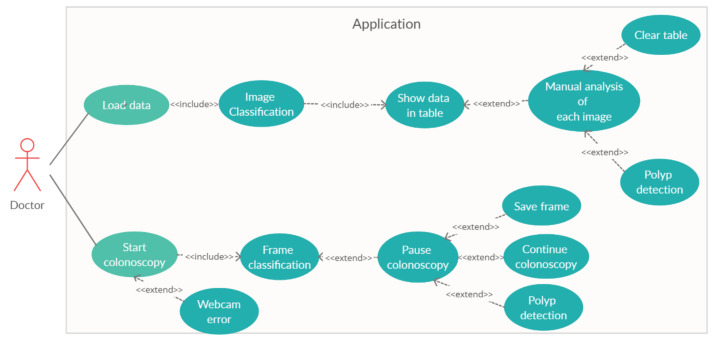
The use-case diagram.

Figure 7 shows the use case diagram of the developed application. The application has two main features. The user can analyse image data, stored in the computer’s memory, or can choose to perform an online colonoscopy.

The most important functions that manage the data flow are presented below.

- The “ImportDataButtonPushed” function is used to load data. It waits until the user has selected one or more images, after which, depending on the number of data entered, prepares the data to be served to the algorithm that deals with tissue classification. Given that the classification operation requires many mathematical operations, the waiting time until the results are ready for display in the table is in the order of seconds. Therefore, an additional window is created that notifies the user that the program has not crashed, but has to wait for a few moments. As soon as the classification is completed, the data are transmitted to the function that handles the information, organising it in a table.
- The “displayResultsIntoTable” function receives as parameters the path to the file, the prediction and the score for each image, and then displays all this infor-mation in a table. In addition, it checks if there are already data in the table, with two options: initialising the table with the new data or concatenating the current ones to the existing ones.
- The “startColonoscopy” function is triggered when the user wants to initiate the colonoscopy process. This establishes the connection to the available camera, in case the connection does not exist or cannot be made for various reasons, a warning message will be displayed. If the connection is successful, a new window will open in which you can view the data received from the webcam and the prediction for that frame will be displayed at the top. At the same time, there will be a button that will allow the user to pause or continue the colonoscopy process. If there is a pause and the frame contains a polyp, then a button will appear that will allow the detection of the malignant area.
- Another important function is “startUpFcn”, which is called upon when launching the application. With this function the variables that will be used are initialised: the network for classification and segmentation, the size of the images. In this way the waiting time is reduced.

### 3.4. Application Facilities

**Figure 8:**
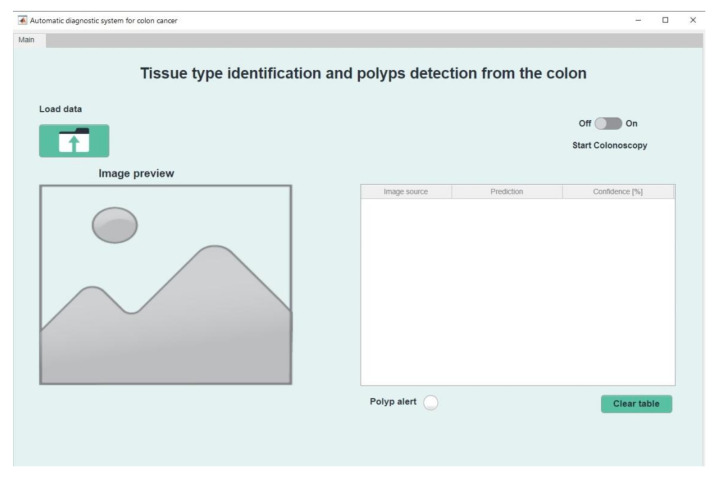
The main menu of the application.

After launching the application, the main window will appear as in Figure 8. Here the main components and functionalities can be seen: a button to load data, another to empty the table, a section where images can be previewed, a switch used to start the colonoscopy process, and an indicator element that serves to signal the presence of a polyp.

**Figure 9:**
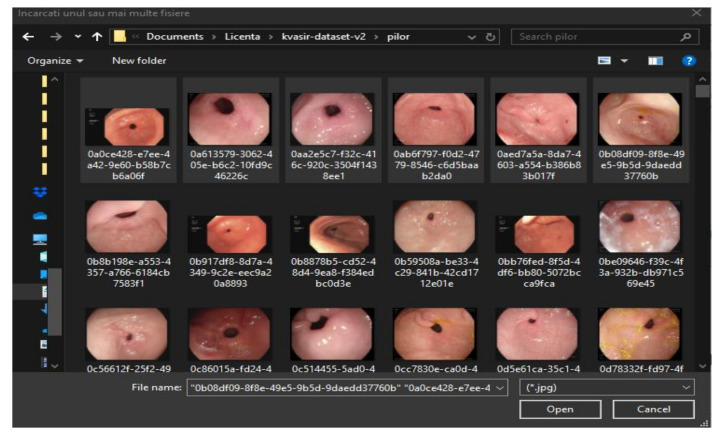
Image loading.

Selecting the top left button will enable data upload. A new window will appear, Figure 9, which allows the navigation through local files and the selection of one or more images. Once the user has decided on the number and desired files, the “Open” button can be pressed.

**Figure 10:**
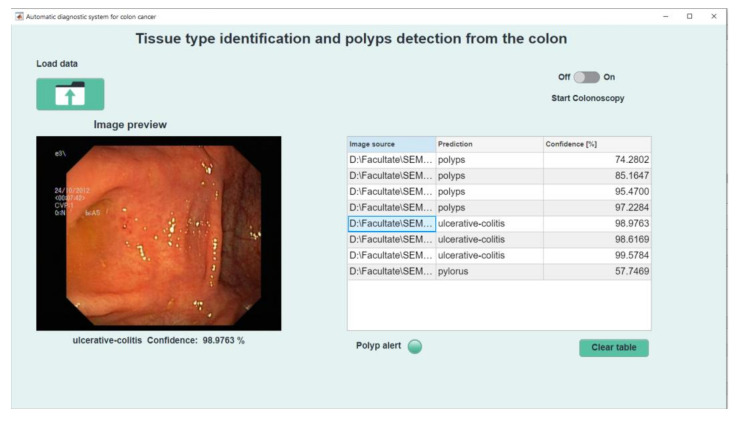
Data preview.

Uploading the data will automatically start the image classification process. It will enter in the table the predictions, the confidence score and the location of the images. When selecting a row in the table, the corresponding image will be loaded in the section intended for preview. Also below this will appear the network prediction and the safety factor on the decision, expressed as a percentage, Figure 10.

**Figure 11:**
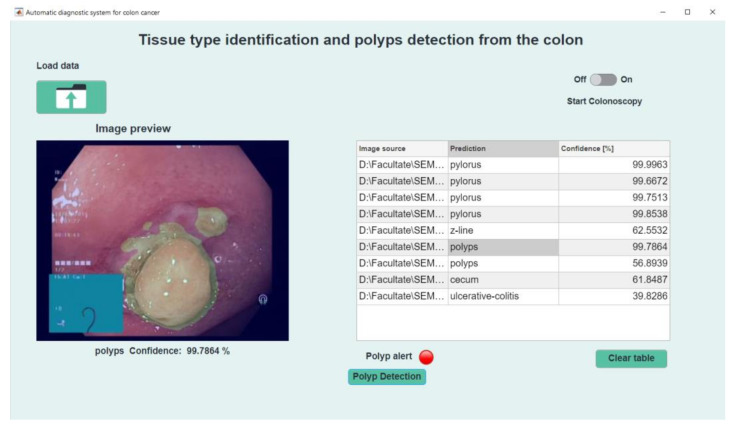
Polyp detection.

If the image selected for analysis contains polyps, then the interface will provide an additional button just below the “Polyp Alert” indicator, called “Polyp Detection”, Figure 11. Pressing this button will lead to image change. In the new image the polyp is highlighted by the yellow tint of the region. By pressing the “Clear table” button and confirming the action, the data will be deleted from the interface and the application will return to the beginning stage.

**Figure 12:**
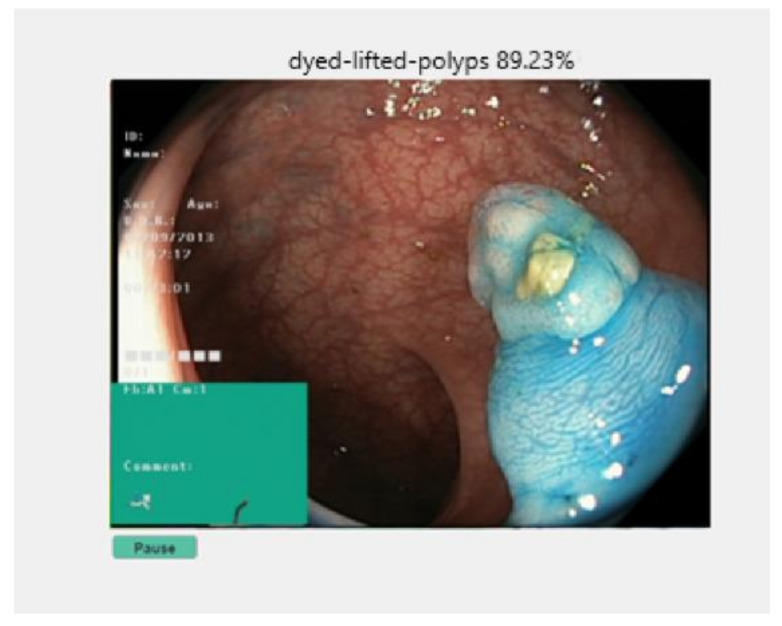
Online colonoscopy.

Another possibility of use is to operate the switch on the top right, called “Start colonoscopy”. It will automatically connect to the available camera. If the connection cannot be made, an error message will appear accompanied by the possible cause. If there are no problems in the connection, the online colonoscopy window appear, Figure 12. At the top of the window is highlighted the network prediction on the frames, and at the bottom is available a “Pause” button.

**Figure 13:**
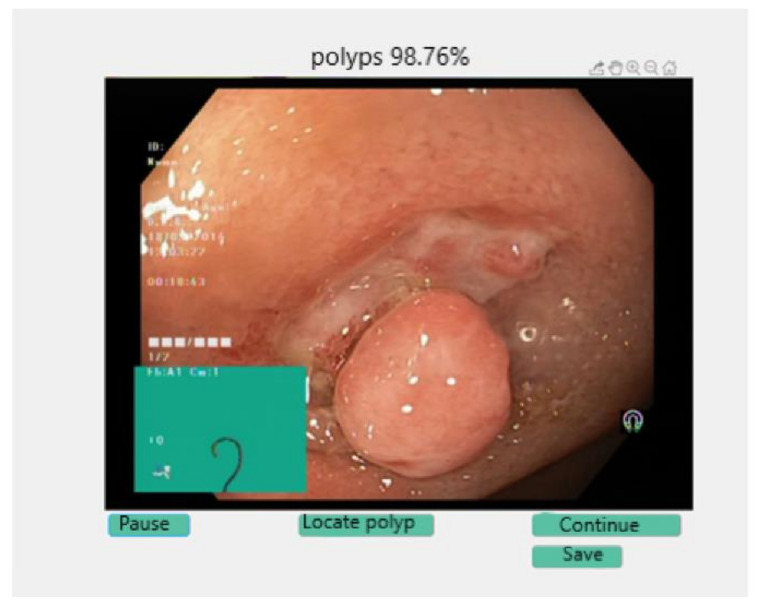
Polyp detected during colonoscopy.

When pressing the “Pause” button, two more buttons will appear that allow the user to resume the colonoscopy or save the current image in the local storage space. In addition, if the frame contains polyps, Figure 13, an additional button appears which locates the malignant area. If the frame on which the process was stopped contains any other class, of the seven remaining available, that button “Locate polyp” is not visible. When pressing the “Continue” button, the process will be resumed, and when select “Save” the image will be saved in the location where the application is installed. If the user closes this window, the application returns to the main window, from where it can resume each step.

## 4. Results

In order to evaluate the results obtained, a confusion matrix is used. This allows easy visualisation of the decisions made by the model and evaluation of the results. From this are extracted the values TP (True Positive), TN (True Negative), FN (False Negative) and FP (False Positive) which will serve as input data for determining the coefficients Sensitivity, Specificity, Accuracy, Precision, F1 Score and Jaccard index, all expressed in percentage [32].

Since this is a medical application, the meaning of each metric must be explained. The interpretation of the results differs according to the intended purpose [42,43,44].

(a) Sensitivity: represents the cases correctly classified as positive relative to the actual number of positive cases:
  (6) $$Sensitivity=\frac{TP}{TP+FN}$$
(b) Specificity: represents the ratio of classified negative cases relative to truly negative cases:
  (7) $$Specificity=\frac{TN}{TN+FP}$$
(c) Precision: means the ratio of those correctly classified as positive to the total number of positive cases:
  (8) $$\;Precision=\frac{TP}{TP+FP}$$
(d) Accuracy: means the ratio of those correctly classified as positive to the total number of positive cases:
  (9) $$Accuracy=\frac{TP+TN}{TP+TN+FN+FP}$$
(e) F1 score: is considered a better benchmark than accuracy when the aim is to compare different models [12]. It is calculated as the harmonic mean of accuracy and sensitivity:
  (10) $$F1=2\frac{Precision\cdot Sensitivity}{Precision+Sensitivity}$$
(j) Jaccard: penalises false detections and it rewards true detection:
  (11) $$J=\frac{TP}{TP+FP+FN}$$

### 4.1. Results Obtained for the “Kvasir” Dataset

The results obtained for the “Kvasir” dataset [30] are presented in Table 4. The training is realised on the second version, which consists of 1000 images for each of the eight classes. The number of data is perfectly balanced for each class. The set is randomly segmented into 70% training data and 30% for validation during the training process, for every 560 iterations. The validation frequency is determined using Equation (12).

(12) $$\nu_{val}=\left[\frac{Number\;of\;Training\;Data}{Number\;of\;Lots}\right]$$

For a better understanding of the impact that the pre-initialised parameters have on performance, the network architecture is taken over and the parameters are reset. Their values are determined using the “Glorot” functions [36], which generate values in a uniform distribution of zero mean and with the variance $\frac{2}{n_{in}+n_{out}}$, where *n_in_* and *n_out_* are calculated according to Equations (13) and (14):

(13) $$n_{in}=FilterSize\left(1\right)\;\cdot \;FilterSize\left(2\right)\;\cdot \;NumChannels$$

(14) $$n_{out}=FilterSize\left(1\right)\;\cdot \;FilterSize\left(2\right)\;\cdot \;NumFilters$$

It is observed that the results obtained in the case of randomly initiated parameters are lower than in other situations. This is expected, moreover it is also concluded in [6,20, 23], while the first layers of networks manage to learn general features for images, such as contours, areas of interest in pictures, shadows, while in depth layers focus on the particularities of each given problem. The “Kvasir” dataset [30] is small compared to “ImageNet” [15] or “Places365” [34], so the trainings made with the parameters obtained on these sets offer superior performance.

Table 5 shows the performances obtained after the data augmentation process. The values of the coefficients are slightly the same as those in Table 4, a surprising fact at a first glance. In papers [9,15,29], the augmentation increased the performance of the model, but in this case the effect is not obtained. The reason is that in the case of the above papers the test data were noisier, the areas of interest were not easily readable, while in the “Kvasir” data set [30] all the data are carefully chosen and thus the areas that are waiting to be identified are clearly visible.

### 4.2. Results for AlexNet

Table 6 highlights the model performances using both the pre-trained model and the one with randomly initialised parameters. The augmentation process of the two situations listed above is also applied. It is also noted that the increase did not improve performance, but there are notable differences between the model with the parameters resulting from the training on the “ImageNet” set [24] and the one in which the parameters were obtained from the training realised only on the “Kvasir” set [30]. The accuracy has a good percentage, but the sensitivity is slightly lower and thus the F1 score is also reduced.

### 4.3. Results for VGG16 and VGG19

In Table 7 are presented the results obtained for *VGG* architecture.

### 4.4. Results for Inceptionv3

Table 8 shows the training results on the dataset “Kvasir” [30]. It can be observed that the metric we are mainly interested in, sensitivity, is about 3–4% higher than in previous cases, even when it was not pre-trained on the “ImageNet” set [24]. The F1 score is also high, indicating a balance between precision and accuracy, in other words high performance.

### 4.5. Results Obtained for Polyp Localisation

In the beginning a different dataset is employed to that used to classify tissue types. The aim was to improve the robustness of the algorithm. The results of training the ResNet18 model on the “CVC-ClinicDB” data set [38] are presented in Table 9.

The dataset augmentation consisted of rotations around the *x* and *y* axes, decreases and enlargements by 30%, and horizontal and vertical cuts with sizes between 0 and 10 pixels. It can be noticed that the values of the Jaccard and F1 coefficients are above the recommended thresholds, so that the model manages to identify the areas of interest quite well. The increase failed to improve performance, which is expected because the data provided do not contain distorted images, but certainly during the endoscopy process, they will be able to bring their input. As endoscopy is a process that takes place in real time, using a video camera, the frames analysed every fraction of a second will be distorted and will capture the areas of interest from different angles. Then the effect of augmentation will be observed by the model’s ability to identify malignant regions. However, evaluating the model on the dataset on which it was made and the network for the part of the classification of tissue types, i.e., on the database called “Kvasir-Seg” [45], Table 10, it can be concluded that performance decreases by about 10%. The reason is the difference between the image types which is specific to each data collection.

**Figure 14:**
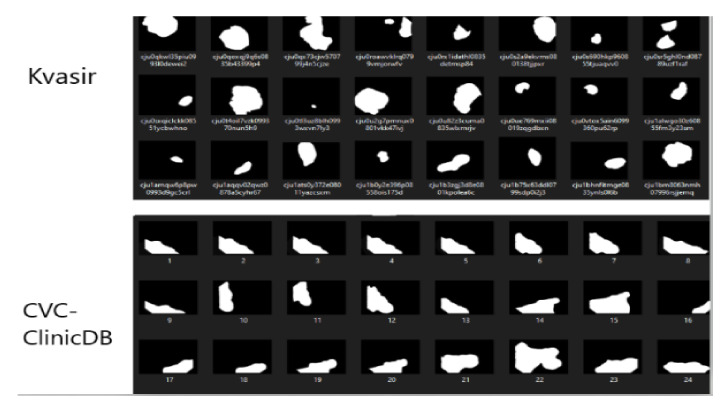
Comparison between different masks of the used dataset.

The images in the “Kvasir-Seg” set [45] are different in terms of shapes and sizes and most importantly are not part of a contiguous sequence of data. On the other hand, the “CVC-ClinicDB” set [38] contains the masks of the images extracted from the video sequences, Figure 14. Therefore the same area appears several times in a row, giving the model the opportunity to use this aspect in its favour to achieve the goal, minimising the error.

To overcome these impediments, the network is also trained on the “Kvasir-Seg” data set [45] using the pre-trained model on “CVC-ClinicDB” [38]. As additional changes to the new set, it is necessary to resize the images to reach the size used for the previous set, and then the image pixels are changed, where they were transformed into values of 0 or 255 corresponding to the tags: tissue or polyp, respectively. The proportions of 60/20/20 regarding the grouping of data for training/validation/testing were kept. The results obtained for the training on the second set, “Kvasir-Seg” [45], and the testing on the 20% of the group reserved for testing in the same set, are presented in Table 11.

In this case the augmentation improved the performance of the model and provided greater robustness due to the fact that the images resulting from the augmentation are more difficult to process and approach the case when frames are extracted during the endoscopy. The Jaccard index, which quantifies the rate of overlap of the predicted and the real area, has a high value, thus suggesting that the model is able to fully identify malignant regions by a proportion of about 75%.

## 5. Conclusions

Artificial intelligence as a concept is increasingly mentioned in everyday discussions in various fields, both those related to technology and in contexts that at first glance do not suggest any connection with this notion (e.g., public administration, management). The sub-field of artificial intelligence is supervised machine learning. In the last decade, many applications have been successfully developed (handwriting recognition, robot control, speech recognition) based on machine learning the performance of which increases in direct proportion to the rate of progress of computing physical resources. Shy, but sure, machine learning has made its presence felt in more sensitive areas, such as the medical field.

The main goal of the present research is to support medical staff, but also patients, with a solution that would help the progress of science and especially improve the chances of healing and recovery of people suffering from colon cancer. With this goal, a colon cancer diagnosis tool was developed. An adjacent goal of this paper is to describe how intelligent algorithms work and to prove their usefulness in the area of prevention and medical diagnosis.

The technological tool which made possible the realisation of this diagnostic system is artificial intelligence. Using specific deep learning techniques, supervised learning and image processing, but also the expertise gained so far by specialists in the field around the globe, we managed to build a model that is able to automatically identify the main types of tissue in the human colon and its specific diseases. Moreover, we have developed an additional model that deals exclusively with the detection of a single type of disease, polyps. The developed tool is able to identify polyps in an image, highlighting them from the rest of the tissues.

To identify the network capable of classifying the eight classes of interest for the topic of this research, we trained and compared five different types of architecture used in typical classification problems. Thus, we identified the most suitable model for the present application. Despite the fact that several metrics for evaluating performance were listed, two of them were finally selected to choose the proper model for this application. Sensitivity and F1 score were the criteria that weighed the most in choosing the network, so *InceptionV3* without augmentation, with a sensitivity of 98.13% and an F1 score of 98.14%, was selected.

In the case of the model for the identification of malignant areas, the metrics used differ from those found in the case of classification. The reason is the difference between the two algorithms. In the first situation we evaluate whether an image is classified correctly as a whole, in the second situation we check the classification at pixel level. Thus, we have the F1 score and the Jaccard index as performance indices. The model trained on the “Kvasir-Seg” set with augmentation was selected in order to be used in the application, because it obtained a Jaccard index of 75.18% and an F1 score of 54.01%.

By assembling the components described above, a desktop application resulted. Its role is to facilitate the interaction between the user, the clinicians, and predictive algorithms. By using the application, the data, in the form of images, are uploaded from the local/external storage space, then they are automatically classified. The user has the possibility to analyze each image and the corresponding prediction. When regions containing polyps appear among the data entered for examination, a button appears on the graphical interface that allows the detection of those malignant areas.

The next step in the research is to include in the diagnosis system the results obtained in [46] by the authors, the computer-aided diagnosis system using an innovative dataset composing of both numeric (blood and urine analysis) and qualitative data (living environment of the patient, tumour position, T, N, M, Dukes classification, associated pathology, technical approach, complications, incidents, ultrasonography-dimensions as well as localisation).

Artificial intelligence still has a lot to offer, its potential being increasingly exploited. Both the medical field and other fields with a direct impact on our lives have the opportunity to progress and provide a superior quality of life as we harmoniously combine our knowledge with the computing power of intelligent algorithms.

## Figures and Tables

**Table 1:** Training options.

Parameter Name	Value
Batch size	10
Number of epoch	10
Learning rate	3 × 10^−4^
Optimisation method	Stochastic Gradient Descent with Momentum
Momentum	0.9

**Table 2:** Training options for semantic segmentation.

Parameter Name	Value
Learn rate drop period	10
Learn rate drop factor	0.3
Initial learn rate	10^−3^
Momentum	0.9
Validation frequency	50
Number of maximum epochs	40
Batch size	2

**Table 3:** Dataset augmentation configuration.

Parameter Name	Value
Pixel range	[−30, 30]
Scale range	[0.7, 1.5]
RandXTranslation	Pixel range
RandYTranslation	Pixel range
RandXshear	[−10, 10]
RandYshear	[−10, 10]
RandXScale	Scale range
RandYScale	Scale range

**Table 4:** GoogleNet training results.

Architecture	Accuracy [%]	Sensibility [%]	Specificity [%]	Precision [%]	F1 [%]
GoogleNet-ImageNet	99.38	97.53	99.65	97.55	97.54
GoogleNet-Places365	98.83	95.3	99.33	95.35	95.32
Uninitialised GoogleNet	96.71	86.85	98.12	87.01	86.93

**Table 5:** GoogleNet training results after augmentation.

Architecture	Accuracy [%]	Sensibility [%]	Specificity [%]	Precision [%]	F1 [%]
GoogleNet-ImageNet	98.44	93.77	99.11	94.04	93.91
GoogleNet-Places365	98.25	93.00	99.00	93.30	93.21
Uninitialised GoogleNet	95.26	81.03	97.29	82.05	81.53

**Table 6:** AlexNet training results.

Architecture	Augmentation	Accuracy [%]	Sensibility [%]	Specificity [%]	Precision [%]	F1 [%]
Pre-initialised AlexNet	-	98.98	94.70	99.24	94.78	94.74
Pre-initialised AlexNet	Yes	97.77	91.08	98.73	91.41	91.24
Uninitialised AlexNet	-	95.59	83.67	97.69	83.85	83.76
Uninitialised AlexNet	Yes	93.58	74.32	96.33	75.39	74.86

**Table 7:** VGG training results.

Architecture	Augmentation	Accuracy [%]	Sensibility [%]	Specificity [%]	Precision [%]	F1 [%]
Pre-initialised VGG16	-	95.03	95.01	99.09	94.09	94.56
Pre-initialised VGG16	Yes	98.41	93.65	99.09	93.95	93.80
Uninitialised VGG16	-	93.53	74.12	96.30	76.24	75.17
Uninitialised VGG16	Yes	91.33	65.30	96.30	76.24	66.37
Pre-initialised VGG19	-	99.10	96.40	99.49	96.54	96.47
Pre-initialised VGG19	Yes	98.02	92.08	98.87	93.34	92.70
Uninitialised VGG19	-	93.34	73.35	96.19	73.24	73.33
Uninitialised VGG19	Yes	91.36	67.66	95.06	67.66	66.53

**Table 8:** InceptionV3 training results.

Architecture	Augmentation	Accuracy [%]	Sensibility [%]	Specificity [%]	Precision [%]	F1 [%]
Pre-initialised Inceptionv3	-	99.53	98.13	99.73	98.15	98.14
Pre-initialised Inceptionv3	Yes	99.41	96.67	99.66	97.67	97.66
Uninitialised Inceptionv3	-	93.98	75.92	96.56	76.07	76.00
Uninitialised Inceptionv3	Yes	92.80	71.45	95.92	73.10	72.27

**Table 9:** Results on “CVC-ClinicDB” dataset.

Augmentation	Accuracy [%]		Jaccard [%]		F1 [%]	
	Per Polyp Tissue Class	Average	Per Polyp Tissue Class	Average	Per Polyp Tissue Class	Average
No	97.1486.59	91.86	95.9367.2	81.56	85.3733.27	59.32
Yes	96.2584.11	90.18	94.7960.66	77.72	84.7828.94	56.86

**Table 10:** Results for “Kvasir” dataset test using the model pre-trained on “CVC-ClinicDB”.

Augmentation	Accuracy [%]		Jaccard [%]		F1 [%]	
	Per Polyp Tissue Class	Average	Per Polyp Tissue Class	Average	Per Polyp Tissue Class	Average
No	89.24 72.70	80.97	84.70 47.08	65.89	71.76 20.33	46.04
Yes	75.81 90.94	83.37	74.44 40.82	57.63	67.94 19.34	43.64

**Table 11:** Results for “Kvasir-seg” dataset.

Augmentation	Accuracy [%]		Jaccard [%]		F1 [%]	
	Per Polyp Tissue Class	Average	Per Polyp Tissue Class	Average	Per Polyp Tissue Class	Average
No	89.76 92.81	91.28	88.5 61.07	74.78	78.530.7	54.6
Yes	89.65 92.95	91.3	88.42 61.94	75.18	78.35 29.68	54.01

## Data Availability

Data supporting reported results can be found on https://datasets.simula.no/kvasir-seg/ (accessed on 5 November 2020) and https://datasets.simula.no/kvasir/ (accessed on 5 November 2020).

## References

[1] Kuipers E.J., Grady W.M., Lieberman D., Seufferlein T., Sung J.J., Boelens P.G., Van De Velde C.J.H., Watanabe T. (2015). Colorectal cancer. Nat. Rev. Dis. Primers.

[2] Haggar F.A., Boushey R.P. (2009). Colorectal Cancer Epidemiology: Incidence, Mortality, Survival, and Risk Factors. Clin. Colon Rectal Surg..

[3] Kainz P., Pfeiffer M., Urschler M. (2017). Segmentation and classification of colon glands with deep convolutional neural networks and total variation regularization. PeerJ.

[4] LeCun Y., Bottou L., Bengio Y., Haffner P. (1998). Gradient-based learning applied to document recognition. Proc. IEEE.

[5] Fleming M., Ravula S., Tatishchev S.F., Wang H.L. (2012). Colorectal carcinoma: Pathologic aspects. J. Gastrointest. Oncol..

[6] Yue X., Dimitriou N., Caie P., Harrison D., Arandjelovic O. (2019). Colorectal Cancer Outcome Prediction from H&E Whole Slide Images using Machine Learning and Automatically Inferred Phenotype Profiles. EPiC Ser. Comput..

[7] Reinhard E., Adhikhmin M., Gooch B., Shirley P. (2001). Color transfer between images. IEEE Eng. Med. Boil. Mag..

[8] Simonyan K., Zisserman A. (2014). Very deep convolutional networks for large-scale image recognition. arXiv.

[9] Urban G., Tripathi P., Alkayali T., Mittal M., Jalali F., Karnes W., Baldi P. (2018). Deep Learning Localizes and Identifies Polyps in Real Time With 96% Accuracy in Screening Colonoscopy. Gastroenterology.

[10] Van Erven T., Harremos P. (2014). Rényi Divergence and Kullback-Leibler Divergence. IEEE Trans. Inf. Theory.

[11] Wang S., Tang C., Sun J., Yang J., Huang C., Phillips P., Zhang Y.-D. (2018). Multiple Sclerosis Identification by 14-Layer Convolutional Neural Network with Batch Normalization, Dropout, and Stochastic Pooling. Front. Neurosci..

[12] Martin M., Sciolla B., Sdika M., Quetin P., Delachartre P. Segmentation of neonates cerebral ventricles with 2D CNN in 3D US data: Suitable training-set size and data augmentation strategies. Proceedings of the 2019 IEEE International Ultrasonics Symposium (IUS).

[13] Sudre C.H., Li W., Vercauteren T., Ourselin S., Cardoso M.J. (2017). Generalised Dice Overlap as a Deep Learning Loss Function for Highly Unbalanced Segmentations. Deep Learning in Medical Image Analysis and Multimodal Learning for Clinical Decision Support.

[14] Dahiru T. (2008). *p*-value, a true test of statistical significance? A cautionary note. Ann. Ib. Postgrad. Med..

[15] Russakovsky O. (2015). ImageNet Large Scale Visual Recognition Challenge. arXiv.

[16] Redmon J., Divvala S., Girshick R., Farhadi A. You only look once: Unified, real-time object detection. Proceedings of the 29th IEEE Conference on Computer Vision and Pattern Recognition, CVPR 2016.

[17] Kang H.-J. Real-Time Object Detection on 640×480 Image with VGG16+SSD. Proceedings of the 2019 International Conference on Field-Programmable Technology (ICFPT).

[18] Xia Y., Cai M., Ni C., Wang C., Shiping E., Li H. A Switch State Recognition Method based on Improved VGG19 network. Proceedings of the 2019 IEEE 4th Advanced Information Technology, Electronic and Automation Control Conference (IAEAC).

[19] Tian X., Chen C. Modulation Pattern Recognition Based on Resnet50 Neural Network. Proceedings of the 2019 IEEE 2nd International Conference on Information Communication and Signal Processing (ICICSP).

[20] Chen C., Qi F. Single Image Super-Resolution Using Deep CNN with Dense Skip Connections and Inception-ResNet. Proceedings of the 2018 9th International Conference on Information Technology in Medicine and Education (ITME).

[21] Tajbakhsh N., Shin J.Y., Gurudu S.R., Hurst R.T., Kendall C.B., Gotway M.B., Liang J. (2016). Convolutional Neural Networks for Medical Image Analysis: Full Training or Fine Tuning?. IEEE Trans. Med. Imaging.

[22] Park D.S. (2016). Colonoscopic polyp detection using convolutional neural networks. Proc. SPIE.

[23] Krizhevsky A., Sutskever I., Hinton G.E. (2012). ImageNet Classification with Deep Convolutional Neural Networks. Proc. Neural Inf. Process. Syst..

[24] Shin Y., Qadir H.A., Aabakken L., Bergsland J., Balasingham I. (2018). Automatic Colon Polyp Detection Using Region Based Deep CNN and Post Learning Approaches. IEEE Access.

[25] Pesapane F., Rotili A., Penco S., Montesano M., Agazzi G., Dominelli V., Trentin C., Pizzamiglio M., Cassano E. (2021). Inter-Reader Agreement of Diffusion-Weighted Magnetic Resonance Imaging for Breast Cancer Detection: A Multi-Reader Retrospective Study. Cancers.

[26] Usuda K., Ishikawa M., Iwai S., Iijima Y., Motono N., Matoba M., Doai M., Hirata K., Uramoto H. (2021). Combination Assessment of Diffusion-Weighted Imaging and T2-Weighted Imaging Is Acceptable for the Differential Diagnosis of Lung Cancer from Benign Pulmonary Nodules and Masses. Cancers.

[27] Debelee T.G., Kebede S.R., Schwenker F., Shewarega Z.M. (2020). Deep Learning in Selected Cancers’ Image Analysis—A Survey. J. Imaging.

[28] Lin T.Y., Maire M., Belongie S., Hays J., Perona P., Ramanan D., Dollár P., Zitnick C.L., Fleet D., Pajdla T., Schiele B., Tuytelaars T. (2014). Microsoft COCO: Common objects in context. Computer Vision–ECCV 2014. ECCV 2014. Lecture Notes in Computer Science.

[29] Bernal J., Sánchez J., Vilariño F. (2012). Towards Automatic Polyp Detection with a Polyp Appearance Model. Pattern Recognit..

[30] Pogorelov K., Randel K.R., Griwodz C., Eskeland S.L., de Lange T., Johansen D., Spampinato C., Dang-Nguyen D.T., Lux M., Schmidt P.T. Kvasir: A Multi-Class Image Dataset for Computer Aided Gastrointestinal Disease Detection. Proceedings of the 8th ACM on Multimedia Systems Conference.

[31] Vrejoiu M.H. (2019). Reţele neuronale convoluţionale, Big Data şi Deep Learning în analiza automată de imagini. Rev. Română Inform. Autom..

[32] Baratloo A., Hosseini M., Negida A., El Ashal G. (2015). Part 1: Simple Definition and Calculation of Accuracy, Sensitivity and Specificity. Arch. Acad. Emerg. Med. (Emerg.).

[33] Szegedy C., Liu W., Jia Y., Sermanet P., Reed S., Anguelov D., Erhan D., Vanhoucke V., Rabinovich A. Going Deeper with Convolutions. Proceedings of the 2015 IEEE Conference on Computer Vision and Pattern Recognition (CVPR).

[34] Zhou B., Lapedriza A., Khosla A., Oliva A., Torralba A. (2018). Places: A 10 Million Image Database for Scene Recognition. IEEE Trans. Pattern Anal. Mach. Intell..

[35] Stacey R. Deep Learning: Which Loss and Activation Functions Should I Use?. https://towardsdatascience.com/deep-learning-which-loss-and-activation-functions-should-i-use-ac02f1c56aa8.

[36] Glorot X., Bengio Y. (2010). Understanding the difficulty of training deep feedforward neural networks. J. Mach. Learn. Res..

[37] Szegedy C., Vanhoucke V., Ioffe S., Shlens J., Wojna Z. Rethinking the Inception Architecture for Computer Vision. Proceedings of the 2016 IEEE Conference on Computer Vision and Pattern Recognition (CVPR).

[38] Bernal J., Sánchez F.J., Fernández-Esparrach M.G., Gil D., Rodríguez C., Vilariño F. (2015). WM-DOVA maps for accurate polyp highlighting in colonoscopy: Validation vs. saliency maps from physicians. Comput. Med. Imaging Graph..

[39] Chen L.C., Papandreou G., Schroff F., Adam H. (2017). Rethinking Atrous Convolution for Semantic Image Segmentation. arXiv.

[40] Szegedy C., Ioffe S., Vanhoucke V., Alemi A.A. Inceptionv4, inception-ResNet and the impact of residual connections on learning. Proceedings of the Thirty-First AAAI Conference on Artificial Intelligence (AAAI-17).

[41] Cui X., Zheng K., Gao L., Zhang B., Yang D., Ren J. (2019). Multiscale Spatial-Spectral Convolutional Network with Image-Based Framework for Hyperspectral Imagery Classification. Remote Sens..

[42] Chen C., Zhou K., Zha M., Qu X., Guo X., Chen H., Wang Z., Xiao R. (2021). An Effective Deep Neural Network for Lung Lesions Segmentation from COVID-19 CT Images. IEEE Trans. Ind. Inform..

[43] Huang Y.-J., Dou Q., Wang Z.-X., Liu L.-Z., Jin Y., Li C.-F., Wang L., Chen H., Xu R.-H. (2020). 3-D RoI-Aware U-Net for Accurate and Efficient Colorectal Tumor Segmentation. IEEE Trans. Cybern..

[44] Tulbure A.A., Tulbure A.A., Dulf E.H. (2021). A review on modern defect detection models using DCNNs–Deep convolutional neural networks. J. Adv. Res..

[45] Jha D., Smedsrud P.H., Johansen D., de Lange T., Johansen H., Halvorsen P., Riegler M. (2021). A Comprehensive Study on Colorectal Polyp Segmentation with ResUNet++, Conditional Random Field and Test-Time Augmentation. IEEE J. Biomed. Health Inform..

[46] Lorenzovici N., Dulf E.-H., Mocan T., Mocan L. (2021). Artificial Intelligence in Colorectal Cancer Diagnosis Using Clinical Data: Non-Invasive Approach. Diagnostics.

